# An alternative way to break the matrix barrier: an experimental study of a LIFU-mediated, visualizable targeted nanoparticle synergistic amplification for the treatment of malignant fibroblasts

**DOI:** 10.3389/fbioe.2024.1486369

**Published:** 2024-11-05

**Authors:** Xiangzhi Zhao, Zhengchao Fan, Junan Zhou, Ying Li, Weiwei Zhu, Song Su, Jizhu Xia

**Affiliations:** ^1^ Department of Ultrasound, The Affiliated Hospital of Southwest Medical University, Luzhou, Sichuan, China; ^2^ Department of Ultrasound, Sichuan Provincial Second Hospital of Traditional Chinese Medicine, Chengdu, Sichuan, China; ^3^ Department of General Surgery (Hepatobiliary Surgery), The Affiliated Hospital of Southwest Medical University, Luzhou, Sichuan, China; ^4^ Department of Plastic and Burns Surgery, The Affiliated Hospital of Southwest Medical University, Luzhou, Sichuan, China

**Keywords:** sonodynamic therapy, Malignant fibroblasts, hematoporphyrin monomethyl ether, ginsenoside RG3, dual-modal imaging, low-intensity focused ultrasound

## Abstract

Malignant fibroblasts (MFs) are widely present in various diseases and are characterized by connective tissue proliferation; these cells act as a physical barrier that severely limits drug delivery and affects disease outcomes. Based on this, we constructed the smart, integrated, theranostic, targeted lipid nanoprobe HMME-RG3@PFH to overcome the bottleneck in the early diagnosis and treatment of MF-related diseases. The protein glucose transporter protein 1 (GLUT-1) is overexpressed on MFs, and its ideal substrate, ginsenoside RG3 (RG3), significantly enhances the targeted uptake of HMME-RG3@PFH by MFs in a hypoxic environment and endows the nanomaterial with stealthiness to prolong its circulation. Perfluorohexane (PFH), a substance that can undergo phase change, was encapsulated in the lipid core and vaporized for ultrasound-enhanced imaging under low-intensity focused ultrasound (LIFU) irradiation. Moreover, hematoporphyrin monomethyl ether (HMME) was loaded into the lipid bilayer for photoacoustic molecular imaging and sonodynamic therapy (SDT) of MFs under the combined effects of LIFU. Additionally, HMME-RG3@PFH instantaneously burst during visualization to promote targeted drug delivery. In addition, the increased number of exposed RG3 fragments can regulate the MFs to enter a quiescent state. Overall, this nanoplatform ultimately achieves dual-modal imaging with targeted and precise drug release for visualization and synergistic amplification therapy, providing a new possibility for the early diagnosis and precise treatment of MF-related diseases.

## Graphical Abstract

Schematic of the design and characterization of the nanoprobe HMME-RG3@PFH.
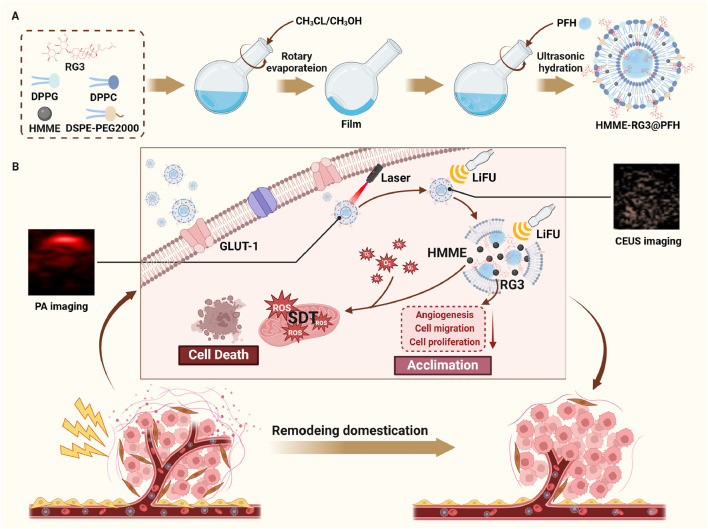

## 1 Introduction

Malignant fibroblasts (MFs) are widely found in solid tumors and are characterized by predominant connective tissue proliferation, organ fibrosis and pathological scarring. Tumors with proliferating fibrous connective tissue, such as those with triple-negative breast cancer (TNBC) and pancreatic ductal adenocarcinoma (PDAC), have bodies rich in collagen fibers, hyaluronic acid, and MFs and form a dense matrix barrier, which severely hinders drug delivery (Miao et al., 2015). Moreover, MFs reduce the immune surveillance abilities of the body and tumor clearance by recruiting immunosuppressive cells. Related studies have shown that the tumor extracellular matrix (ECM) is remodeled by proteases [e.g., hyaluronidase (Jacobetz et al., 2013) and collagenase (Zinger et al., 2019)] to disrupt the physical barrier (Fu et al., 2022) and the normal tissue ECM, which in turn accelerates cancer cell metastasis. Organ fibrosis accounts for a relatively large proportion of fibrotic diseases (Zhao et al., 2020; Nanthakumar et al., 2015). MFs form myofibroblasts by upregulating the expression of fibrotic cytokines, which cannot undergo apoptosis (Mascharak et al., 2022), ultimately leading to the excessive deposition of ECM, which induces structural disorders in organs and subsequent loss of function (Tomasek et al., 2002). Currently, the clinical outcome after organ fibrosis is poor. In addition, keloid skin disease is often accompanied by abnormal fibroblast activity and significant vascularization; this abnormal wound healing process can lead to organ dysfunction, deformity, and even serious mental health concerns (Yuan et al., 2019). In recent years, the incidences of hyperplastic keloids and keloids have has risen markedly, and the affected population has tended to be younger, becoming an enormous medical burden on society. More than 100 million new cases are reported annually in developed countries alone (Bayat et al., 2003), and the relapse rate is high after conventional treatments. MFs play an important role in the development of many diseases, but their clinical therapeutic efficacy is unsatisfactory, and they face great challenges. Therefore, the regulation and reduction of MFs has become a popular but difficult topic in current research as a new strategy for treatment with the potential for development.

In recent years, sonodynamic therapy (SDT) has overcome the photodynamic limitations due to its advantages in terms of noninvasiveness, targeting and ease of operation (Yue et al., 2019). Moreover, focused ultrasound has been adopted to mediate the destruction of targeted microbubbles, which effectively increases the permeability of cell membranes and the capillary interstitial space for deep and balanced drug distribution. Compared with first generation acoustic sensitizers, which have many disadvantages, hematoporphyrin monomethyl ether (HMME) has the advantages of easy preparation, stability, low dark toxicity and strong acoustic excitation absorption (Son et al., 2020; Wen et al., 2022; Xu et al., 2020). However, the hydrophobicity of HMME makes it prone to agglomeration, which reduces its bioavailability. In contrast, lipid materials are regarded as ideal materials in many fields, such as drug delivery and molecular imaging, due to their high stability and biocompatibility, and combining lipids with HMME can allow the limitations of HMME to be overcome.

Ginsenoside RG3 (RG3) is an amphiphilic material possessing a hydrophilic sugar group and a lipophilic steroidal structure similar to cholesterol that reduces the risk of distant invasion and *in situ* metastasis by modulating neovascularization (Zeng et al., 2022; Nakhjavani et al., 2020). Therefore, RG3 can be loaded into nanodrug carriers to replace cholesterol. In addition, RG3 specifically recognizes glucose transporter protein 1 (GLUT-1) on the MF cell membrane to improve active targeting. The application of RG3 to fibroblasts and in tumor-related experiments has been reported with somewhat low frequency and has mostly been delivered via a single mode. Some undesirable physicochemical properties, such as its hydrophobicity and short blood half-life, lead to the saponin RG3 having low bioavailability and poor efficacy, which are the main reasons that limit its clinical application; however, using lipid nanocarriers can overcome the bottlenecks of drug depletion and drug release at nontarget sites.

Under ultrasound irradiation, perfluorohexane (PFH) undergoes phase change and is converted into microbubbles (MBs) by acoustic droplet vaporization (ADV) for ultrasound imaging and local targeted drug release. Photoacoustic imaging does not require ionization, which overcomes the depth limitations of traditional optical imaging, and possesses the advantages of high spatial resolution and specificity for functional imaging. These two imaging methods complement each other. Moreover, molecular ligands targeting MFs can be used for visualization in terms of the early diagnosis of lesions and precise targeted drug release.

In this work, the targeted lipid nanoprobe HMME-RG3@PFH was constructed. The membrane adjuvant modification strategy of replacing cholesterol with RG3 was adopted to improve the nanoprobe’s circulation time and reduce macrophage immunosurveillance, while the encapsulated HMME and PFH enabled photoacoustic/ultrasound dual-modal imaging. The active targeting properties of RG3 combined with passive targeting by the enhanced permeability and retention (EPR) effect enhanced the efficient homing of the nanoprobes to MFs, thereby improving the early diagnosis of diseases of this class. Therapeutically, due to the dual targeting effect of HMME-RG3@PFH, the nanomaterial efficiently accumulated locally in the lesion. When used in combination with low-intensity focused ultrasound (LIFU) for dual-modal imaging, HMME-RG3@PFH achieved precise drug release and SDT, while RG3 regulated MFs and remodeled the target microvessels to produce synergistic therapeutic effects. Overall, HMME-RG3@PFH is an ideal drug delivery material for the future clinical treatment of MF-related diseases.

## 2 Materials and methods

### 2.1 Materials

Dipalmitoyl phosphatidylcholine (DPPC), distearoyl phosphatidylglycerol (DPPG), distearoyl phosphatidylethanolamine-polyethylene glycol 2000 (DSPE-PEG2000), and cholesterol (CHOL) were purchased from Ruixi Biotechnology Co. Ltd. (Xi’an, China). PFH was purchased from Aladdin Biochemistry & Technology Co. Ltd. (Shanghai, China). HMME was purchased from MacLean Biochemistry & Technology Co. Ltd. (Shanghai, China). RG3 was obtained from Efar Biotech Co. (Chengdu, China). 4′,6-Diamidino-2-phenylindole (DAPI), 2,7-dichlorofluorescein diacetate (DCFH-DA) and the cell membrane far-infrared fluorescent probe DiD were purchased from BiyunTian Biotechnology (Shanghai, China). A calcein acetoxymethyl/propylene iodide (calcein-AM/PI) kit was purchased from Solepol Science and Technology Ltd. (Beijing, China). Keloid fibroblasts (KFs) were selected as representative MFs for the experiments and were obtained from Guangzhou Mingan Biotechnology Co., Ltd. RAW264.7 macrophages and human umbilical vein endothelial cells (HUVECs) were obtained from the Laboratory of Oncology Radiobiology, Affiliated Hospital of Southwest Medical University.

### 2.2 Methods

#### 2.2.1 Preparation of HMME-RG3@PFH

The nanoprobe HMME-RG3@PFH was prepared by thin film dispersion-ultrasonic emulsification. First, the lipid materials and prodrugs were weighed in a certain ratio (DPPC:DSPE-PEG2000:DPPG:RG3:HMME = 5:1.5:2:2:2) and dissolved in organic solvents. Then, the above mixture was subjected to rotary evaporation until a homogeneous film formed, and the film was dissolved in PBS under ultrasound to form a homogeneous semitransparent red aqueous emulsion. Next, 100 µL of PFH was added to an ice bath sonicator (power: 120 W; time: 5 min; on: 4 s; off: 5 s), and centrifugation was performed three times at low temperature to obtain HMME-RG3@PFH. The steps to synthesize the RG3@PFH and HMME-CHOL@PFH nanoparticles were similar to those described above, blank nanocarriers were prepared without the addition of HMME or RG3, and the fluorescently labeled nanoparticles were prepared by adding DiD after film resuspension. All procedures were carried out under protection from light.

#### 2.2.2 Characterization of HMME-RG3@PFH

Transmission electron microscopy was used to observe the nanomorphology of HMME-RG3@PFH; the hydrated particle size, dispersion coefficient and zeta potential were determined by a laser particle size potential analyzer after dilution; and samples were taken at different times (1, 2, 3, 4, 5, 6, and 7 days) to determine the average particle size and dispersion coefficient of HMME-RG3@PFH. A methanol film breaking assay was used to determine the drug content in HMME-RG3@PFH. Briefly, the nanoparticles were resuspended in methanol and sonicated with ultrasound if necessary, after which the supernatant was collected by centrifugation. The encapsulation efficiency (EE%) and drug loading rate (LE%) of HMME and RG3 were determined by UV spectrophotometry and high-performance liquid chromatography, respectively, using the following equations:

$$\text{EE}\%=\left(W1/W2\right)\times 100\%\text{ LE}\%=\left(W1/W3\right)\times 100\%$$

where W1 is the mass of the drug in the nanoparticle, W2 is the mass of the drug added, and W3 is the total mass of the nanoparticles.

#### 2.2.3 Drug release

Equal amounts of the HMME-RG3@PFH nanoparticle formulation were dissolved in PBS and placed in dialysis bags (MW: 3.5 kDa). One group was irradiated (5 W/cm^2^, pulse mode, 3 min), and the other was not. Then, the bags were placed in 40 mL of dialysis buffer containing PBS and 0.1% Tween 80 (pH = 6.5) with continuous shaking on a thermostatic shaker (37°C, 120 rpm). At different time points, 2 mL of dialysis buffer was removed and stored at 4°C away from light, an equal amount of fresh buffer was added back to the system, and the amount of HMME released into the dialysate was analyzed spectrophotometrically. Finally, the cumulative release curve was plotted.

#### 2.2.4 Ultrasound imaging and phase change capability

An agar model with a 3% mass concentration was configured, and HMME-RG3@PFH solution (0.2 mg/mL, 200 µL) was added to the agar model for ultrasound (US) and contrast-enhanced ultrasound (CEUS) assessments under different powers and for different irradiation durations (relevant parameters: 3–6 W/cm^2^, pulse mode, 1–4 min). US and CEUS images were captured before and after irradiation using an ultrasonic diagnostic instrument, and the relevant signal intensity was analyzed. Optical microscopy was used to observe the phase transition of HMME-RG3@PFH before and after LIFU irradiation (parameters: 5 W/cm^2^, pulse mode, 1–5 min).

#### 2.2.5 Photoacoustic imaging

200 µL HMME-RG3@PFH nanoparticle solutions at different concentrations (1.2 mg/mL, 2.4 mg/mL, 3.6 mg/mL, 4.8 mg/mL, 7.2 mg/mL, and 9.6 mg/mL) were added to agar gel wells, and an equal amount of PBS was added between neighboring wells to reduce interference. The, the samples were irradiated with a laser in the wavelength range of 680 nm–970 nm, and the photoacoustic signals were captured by a photoacoustic imaging system.

#### 2.2.6 Assessments of cellular uptake and targeting

Logarithmically grown RAW264.7 cells and KFs were inoculated in 6-well plates at a density of 1.0 × 10^4^/well and incubated for 24 h at 37°C in a 5% CO_2_ environment. Fresh solutions of DiD-labeled HMME-RG3@PFH and HMME-CHOL@PFH at a concentration of 50 μg/mL were incubated with RAW264.7 cells and KFs for 4 h. Then, the solution was aspirated, and the cells were washed 3 times with PBS, fixed with 4% paraformaldehyde for 15 min and then washed again with PBS 3 times. Then, 0.5 mL of DAPI solution was added to each well with shaking for 5 min to uniformly stain the cells. Afterward, the fluorescence was observed by fluorescence microscopy.

KFs and HUVECs were inoculated at a density of 1.0 × 10^5^/well and incubated at 37°C with 5% CO_2_ for 24 h until the cells had completely adhered to the wall. Then, the medium was removed and DiD-labeled HMME-RG3@PFH at a concentration of 50 μg/mL was added, and the cells were placed in the incubator mentioned above for incubation for 1 h, 2 h, or 4 h protected from light. Afterward, the medium was removed, and the cells were washed three times with PBS. Then, 0.5 mL of trypsin solution was added to each well, and after digestion was terminated, centrifugation was performed three times (1,000 rpm, 2 min). The cells were then resuspended in 0.5 mL of PBS, and finally, the nanoparticle cellular uptake efficiency was assessed by flow cytometry.

#### 2.2.7 Cell proliferation assay

KFs grown to the logarithmic phase were cultured with complete DMEM at 37°C and 5% CO_2_, digested with trypsin when the cell density reached 80%–90%, resuspended, centrifuged, and spread uniformly in 96-well plates at a density of 1.0 × 10^4^/well. After 24 h, 100 µL of blank lipid nanoformulation culture medium was added to the plates, and after 24 h of coculture, the MTT solution was added. After incubation at 37°C for 4 h, zymography was used to detect the absorbance (OD) at 490 nm.

KFs were spread into 96-well plates at a density of 1.0 × 10^4^/well using a procedure similar to that described above and incubated at 37°C with 5% CO_2_ for 24 h. Afterward, the cells were randomly divided into the following five groups: control, LIFU irradiation, RG3@PFH, HMME-RG3@PFH, and HMME-RG3@PFH + LIFU. After 24 h, the cells had completely attached to the wall, and the medium was removed and replaced with 100 µL of fresh medium containing the test material for an additional 24 h of incubation under the same conditions. And, LIFU irradiation was applied to the appropriate groups (LIFU and HMME-RG3@PFH + LIFU group) after 4 h of incubation (5 W/cm^2^, pulse mode, 3 min). Then, the MTT solution was added, and the cells were incubated for another 4 h before the survival rates were calculated, as follows: (OD value of the experimental group - OD value of the blank group)/(OD value of the control group - OD value of the blank group) × 100%. Blank lipid nanoparticles were added in the LIFU irradiation group. Nanoparticles were not added to the control group, and the blank group did not contain nanoparticles or cells. Analysis of HUVECs was performed using experimental methods similar to those described above.

#### 2.2.8 Wound healing experiments

HUVECs were cultured in 6-well plates at a density of 2.0 × 10^5^/well, the cells in the center of the dish was scraped with a sterile pipette tip after the cells had grown to 80%–90% confluence, and the cells at the scratched areas were washed with PBS. The nanoparticles were dissolved in DMEM (at a concentration of 50 μg/mL), added 1 mL to the corresponding wells of the plates, each well plate was then irradiated with LIFU (5 W/cm^2^, pulse mode, 3 min), and cultured at 37°C in a 5% CO_2_ incubator for 24 h. Cell migration was recorded by microscopy at 0 and 24 h. ImageJ software was used to analyze scratch areas, and the cell scratch healing rate was calculated as follows: (0 h area - 24 h area)/0 h area × 100%.

#### 2.2.9 Transwell experiments

The substrate gel was diluted 1:10 and precooled before being evenly applied to the bottom of the upper Transwell chamber. The chamber was placed in a 37°C, 5% CO_2_ incubator for 4 h, after which the excess culture medium was removed along the edges of the chamber. KFs that had been starved for 12 h were inoculated at a density of 2 × 10^4^/well in the upper chamber of the Transwell and cultured in serum-free DMEM. Add 100 μL DMEM diluted nanoparticles of each component to the corresponding well plate in the upper chamber (concentration 50 μg/mL). Additionally, 600 µL of DMEM culture medium containing 20% fetal bovine serum was added to the bottom chamber, the cells were irradiated (5 W/cm^2^, pulse mode, 3 min) and cultured for 24 h. Then, the cells in the upper chamber were removed with cotton swabs, and the cells in the lower chamber were fixed with 10% paraformaldehyde, stained with 0.1% crystal violet for 20 min, and observed under a microscope. Four fields of view were randomly selected for each sample, and cell counting was performed with ImageJ software.

#### 2.2.10 Calcein-AM/PI fluorescence staining

KFs were inoculated in six-well plates at a density of 1.0 × 10^5^/well and randomly divided into the following five groups: control, LIFU, RG3@PFH, HMME-RG3@PFH, and HMME-RG3@PFH + LIFU. The cells were cultured for 24 h at 37°C in an incubator. When the cell density reached 70%–80%, add the appropriate solution to each well (at a concentration of 25 μg/mL), and the cells in the HMME-RG3@PFH + LIFU and LIFU groups were irradiated after 4 h of incubation (5 W/cm^2^, pulse mode, 3 min). After 24 h of coculture, the culture medium was removed, and calcein-AM/PI solution was added to each well, after which the plates were incubated for 20 min in the dark. Afterward, the unbound dye was removed by washing the cells with PBS, and the plates were placed under an inverted fluorescence microscope for observation.

#### 2.2.11 Cytotoxic reactive oxygen species (ROS)

KFs were inoculated in six-well plates at a density of 1.0 × 10^4^/well and cultured at 37°C with 5% CO_2_ for 24 h. Afterward, the cells were randomly divided into the following five groups: control, LIFU, RG3@PFH, HMME-RG3@PFH, and HMME-RG3@PFH + LIFU. Test solutions with the appropriate components at a concentration of 50 μg/mL were added to each well, and the cells in the LIFU and HMME-RG3@PFH + LIFU groups were subjected to ultrasonic irradiation after 4 h of incubation (5 W/cm^2^, pulse mode, 3 min). Next, the cells were washed with PBS three times, and DCFH-DA solution (prepared in serum-free medium and diluted 1:1,000) was added. The well plate was placed in a 37°C, 5% CO_2_ incubator in the dark to allow staining for 30 min, after which an inverted fluorescence microscope was used to observe ROS production.

#### 2.2.12 Hemolysis test

Whole blood was obtained from the veins of healthy BALB/C mice and collected in EDTA-containing anticoagulation tubes, mixed well and centrifuged (3,000 rpm, 20 min). Then, 1 mL of HMME-RG3@PFH solution at different concentrations (25 µg/mL–200 μg/mL), ultrapure water, and 0.9% saline were mixed with 30 µL of blood cells for incubation at 37°C in a thermostatic water bath for 4 h followed by centrifugation (3,000 rpm, 10 min). After centrifugation, the samples were photographed, and the supernatant was removed with a pipette. Then, the absorbance of the supernatant at 540 nm was measured with a spectrophotometer, and the corresponding hemolysis rate was calculated as follows: $\text{hemolysis rate }\left(\%\right)=\left(\text{OD value of the sample}-0.9\%\text{ OD value of saline}\right)/\left(\text{OD value of ultrapure water}-0.9\%\text{ OD value of saline}\right)\times 100\%$ . After performing the procedure described above, the morphology and structure of the erythrocytes were observed under a light microscope.

#### 2.2.13 Pathological scar modeling

All animal studies followed the guidelines for the use of experimental animals and were approved by the Animal Experiment Center of Southwest Medical University. Adult New Zealand large white rabbits (male, 1.5–2 kg) were anesthetized with 1% sodium pentobarbital (1 mg/kg). Six full-layer wounds were created ventrally in each ear using a 10-mm perforated biopsy tool under sterile conditions. The epidermis, dermis and cartilaginous membrane were excised using a surgical scalpel and routinely disinfected postoperatively, followed by re-disinfection of the wounds and removal of the wound secretions the next day.

#### 2.2.14 *In vivo* therapy

A rabbit ear pathological wound model was used to study the antifibrotic effect of the HMME-RG3@PFH nanoparticles. At 28, 35, and 42 days after surgery and after complete epithelialization, the wounds were randomly divided into the following five groups: control, IH-HMME-RG3@PFH, IH-HMME-RG3@PFH + LIFU, IV-HMME-RG3@PFH + LIFU, and IH-5-FU, in which the control group was injected with an equal volume of 0.9% saline. Briefly, HMME-RG3@PFH was dissolved in 0.9% saline, and 0.1 mL of the HMME-RG3@PFH solution (2 mg/mL) was injected into the subcutaneous tissue.

#### 2.2.15 Scar assessment and histological analysis

Photographs were taken to observe the gross morphology of the scars, and specimens were collected 49 days after injury for histological analysis. The most significant part of the tissue scar was selected and fixed in 4% paraformaldehyde for 24 h. After embedding in paraffin, the specimens were cut into 5 µm sections, fixed on slides, and subjected to Masson staining. Then, the dermis from each of the five groups was randomly selected and photographed.

### 2.3 Statistical analysis

Statistical analysis was performed using GraphPad Prism 10. Two-by-two comparisons were performed using t tests, one-way ANOVA was used for between-group comparisons, and variable correlation was analyzed by linear correlation analysis. A p-value <0.05 was considered to indicate statistical significance.

## 3 Results and discussion

### 3.1 Characterization of HMME-RG3@PFH

**FIGURE 1:**
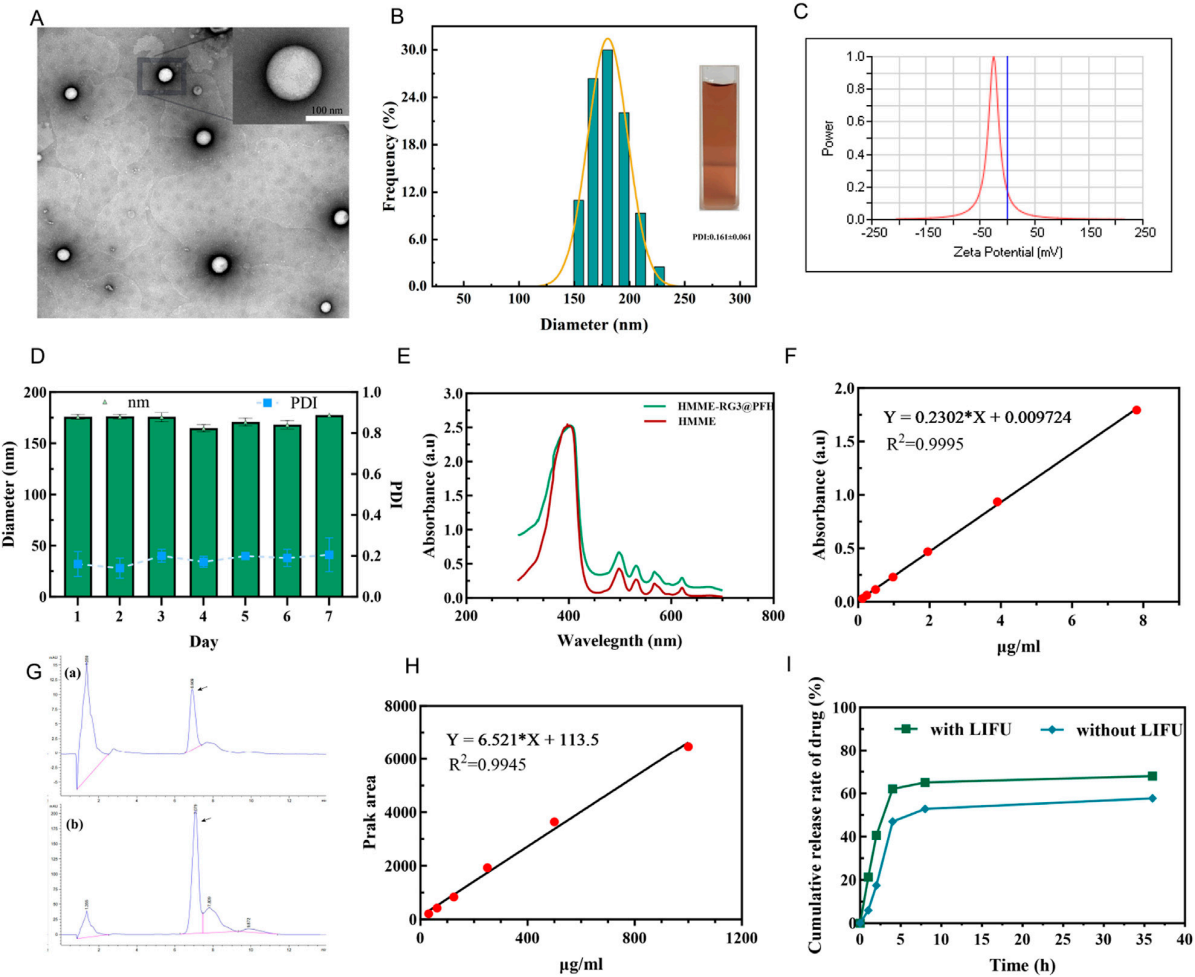
Characterization of the HMME-RG3@PFH nanoprobe. **(A)** Transmission electron microscopy image of HMME-RG3@PFH; scale bar: 100 nm. **(B)** Nanoparticle size distribution. **(C)** Nanoparticle zeta potential value. **(D)** Changes in the particle size and dispersion index in serum. **(E)** UV spectra of HMME and HMME-RG3@PFH. **(F)** Standard curve for HMME. **(G)** a and b are the high-performance liquid chromatography chromatograms of RG3 and HMME-RG3@PFH. **(H)** RG3 peak area versus concentration standard curve. **(I)** Cumulative HMME release with and without LIFU irradiation (pH = 6.5).

Transmission electron microscopy revealed that the nanoparticles were homogeneous and spherical with smooth and well-dispersed surfaces (Figure 1A), and the average particle size was 175.93 ± 2.27 nm (PDI: 0.161 ± 0.061) and uniform, as detected by a particle size analyzer (Figure 1B). This would reduce the clearance by hepatic and renal tissues and allow passive targeting via the EPR effect (Peng et al., 2020; Hong et al., 2020; Ikeda-Imafuku et al., 2022). The zeta potential was −26.88 ± 1.59 mV (Figure 1C), and such a negative potential can reduce the interactions between the nanoparticles and proteins in circulation and the protein corona, which can interfere with nanoparticle circulation (Du et al., 2018). The nanoparticles were stored for 7 days at 4°C without any significant change in size or PDI (Figure 1D), which indicated good physical stability due to the formation of a hydration layer from the hydrophilic sugar chain of RG3 and synergistic modification with functionalized PEG. The UV spectrum showed a maximum absorption peak at 400 nm in the HMME sample (Figure 1E), while the HMME-RG3@PFH nanoparticles also showed a corresponding absorption peak at 400 nm, indicating that HMME was successfully encapsulated in HMME-RG3@PFH. A standard curve of HMME was constructed using the absorbance values (Figure 1F), and values of 61.82% for encapsulation and 10.30% for drug loading were obtained for HMME in HMME-RG3@PFH. Using high-performance liquid chromatography, the same characteristic peaks appeared in the RG3 and HMME-RG3@PFH spectra (Figure 1G), indicating that HMME-RG3@PFH contained RG3. Based on the corresponding peak area standard curves (Figure 1H), the percentage of RG3 encapsulated in HMME-RG3@PFH was 73%, and the drug loading rate was 9.12%. Quantitative analysis of the release pattern of HMME from HMME-RG3@PFH revealed that after 2 h of irradiation, the amount of drug released in the LIFU-irradiated group (40.55%) was significantly greater than that in the unirradiated group (17.49%). Moreover, release from HMME-RG3@PFH was more stable after 8 h of LIFU irradiation (release rate of 65.05%), which was greater than the 52.75% release rate in the unirradiated group. This result was attributed to the rapid drug release that occurred after ADV due to LIFU irradiation (Figure 1I).

### 3.2 Ultrasound/photoacoustic imaging

**FIGURE 2:**
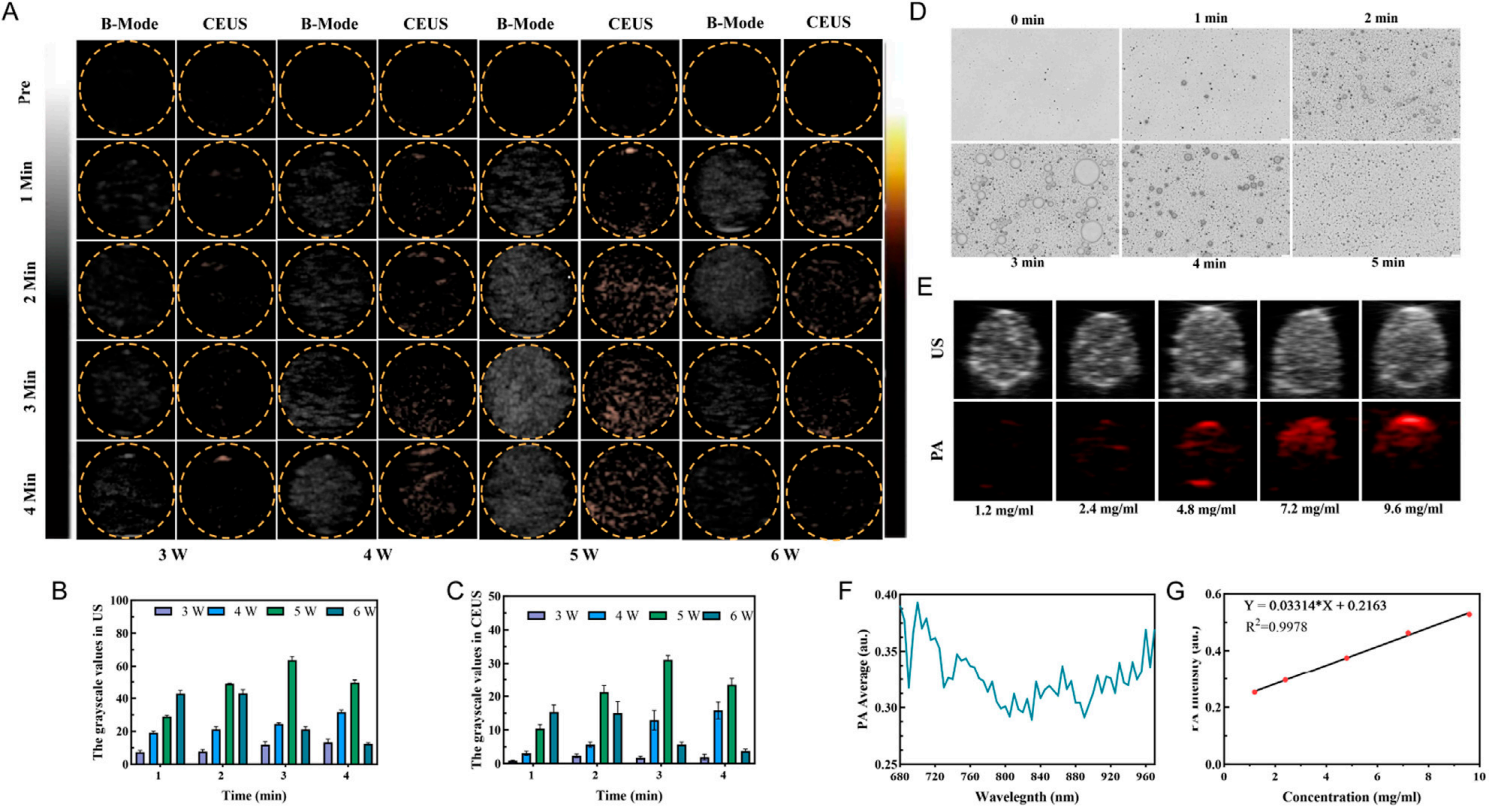
HMME-RG3@PFH imaging characteristics. **(A)** US mode and CEUS mode images of HMME-RG3@PFH before and after LIFU irradiation under different conditions (3–6 W/cm^2^, 1–4 min). **(B)** Analysis of the US signals. **(C)** Analysis of the CEUS signals. **(D)** Photomicrograph images of HMME-RG3@PFH before and after LIFU irradiation (5 W/cm^2^, 1–5 min). **(E)** US and PA images of solutions of HMME-RG3@PFH at different concentrations. **(F)** Photoacoustic signal intensity of the HMME-RG3@PFH nanoparticles at wavelengths from 680 nm to 970 nm. **(G)** Standard curve of HMME-RG3@PFH PA signal intensity.

The ADV effect of PFH was evaluated using ultrasound imaging to determine the optimal ultrasound power and irradiation duration, which are important for the development of subsequent therapeutic strategies for this synergistic amplification nanoplatform. We explored the effect of LIFU irradiation power and duration (3–6 W/cm^2^ and 1–4 min) on the phase transition and found that at a power of 3 W/cm^2^, the signal did not change due to a change in structural stiffness. Although the US and CEUS signals were initially weak, they increased gradually with time as the irradiation power increased (Figure 2A), showing a large increase at 5 W/cm^2^. However, at a power of 5 W/cm^2^, the signal decayed at 4 min, significant signal degradation was also found at 6 W/cm^2^, which was mainly due to collapse and structural instability caused by microbubble polymerization. Additionally, the acoustic phase transition of the nanoprobe HMME-RG3@PFH was power and time dependent, giving a signal at 5 W/cm^2^ and 3 min. All the signal intensities were weak at 3 W/cm^2^ for 1–4 min, but gradually increased with time at 4 W/cm^2^, and were the strongest at 5 W/cm^2^ after 3 min of irradiation (Figures 2B, C). Therefore, 5 W/cm^2^ for 3 min was selected as the parameter to maximize the ADV effect.

After LIFU irradiation, HMME-RG3@PFH transformed into microbubbles, which reached a maximum density after 3 min of irradiation (Figure 2D). After this time, adjacent microbubbles fused with each other and converged into large microbubbles until they ruptured. The above results indicated that HMME-RG3@PFH had good imaging performance, which was attributed to excitation of the PFH in the core by LIFU to produce an ADV effect, and the strong acoustic impedance difference-induced backscattered signal enabled ultrasound imaging (Zhu et al., 2019). This result is consistent with the peak intensity at 3 min from *in vitro* ultrasound imaging, which provides a solid experimental basis for the subsequent selection of the LIFU irradiation time.

Further exploring the photoacoustic imaging effect of the nanoprobe HMME-RG3@PFH due to the acoustic sensitivity of HMME, a signal at 700 nm was observed in the excitation wavelength range from 680 nm to 970 nm (Figure 2F). Photoacoustic signals were observed with 2.4 mg/mL HMME-RG3@PFH, which gradually increased in intensity with increasing concentration to 9.6 mg/mL, where the signals were significantly enhanced (Figures 2E, G). These data show that the drug concentration at the target area is expected to be sufficient to enable visual diagnosis and treatment.

### 3.3 Phagocytosis and targeted uptake

The long circulation and targeting abilities of HMME-RG3@PFH were of great interest in this study and are also key to targeted imaging and cell therapy. Therefore, this experiment is divided into two parts: in the first part, the immune escape ability of the nanoparticles under macrophage surveillance was observed, and in the second part, the targeted uptake of nanoparticles by KFs was evaluated.

**FIGURE 3:**
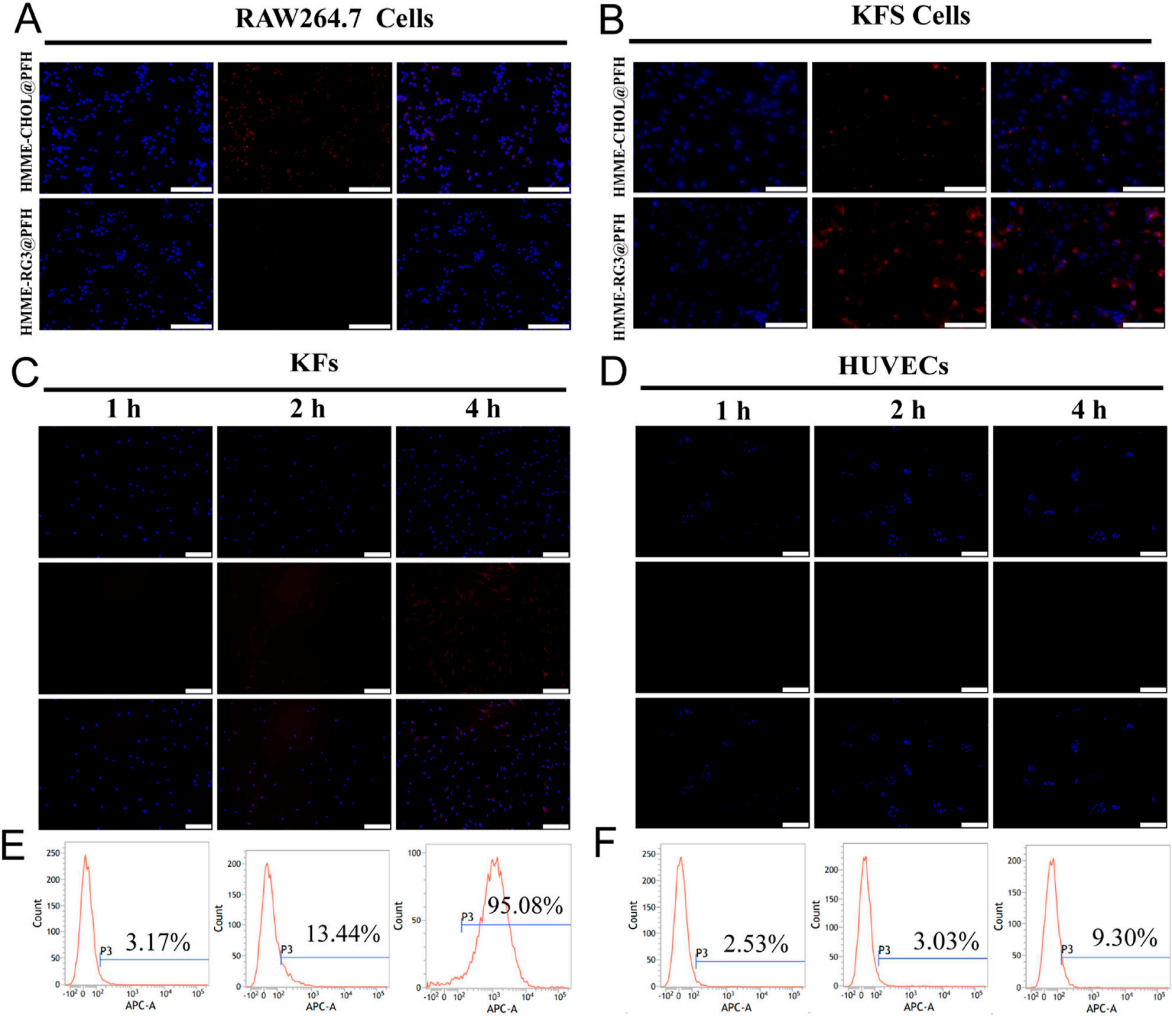
Targeted cellular uptake. **(A)** Fluorescence images of RAW264.7 cells coincubated with HMME-RG3@PFH and HMME-CHOL@PFH (DAPI-stained nuclei in blue, DiD-labeled nanoparticles in red); scale bar: 100 µm. **(B)** Fluorescence images of KFs coincubated with HMME-RG3@PFH and HMME-CHOL@PFH; scale bar: 100 µm. **(C)** Plots of the cellular uptake of HMME-RG3@PFH by KFs after different durations; scale bar: 200 µm. **(D)** Plots of the cellular uptake of HMME-RG3@PFH by HUVECs after different durations; scale bar: 200 µm. **(E)** Rate of cellular uptake of HMME-RG3@PFH by KFs. **(F)** Uptake of HMME-RG3@PFH by HUVECs.

Highly bioavailable drugs need to have long circulation times, and carriers play an important role in preventing drugs from being rapidly recognized and cleared by the phagocytosis system (Di et al., 2021). Macrophages, as a constituent part of the mononuclear phagocyte system, are mainly responsible for the recognition and clearance of foreign substances; therefore, these experiments were designed to investigate the effect of RAW264.7 macrophages on the uptake of fluorescent HMME-RG3@PFH versus their uptake of fluorescent HMME-CHOL@PFH. More HMME-CHOL@PFH was taken up after coincubation; in contrast, the fluorescence intensity due to HMME-RG3@PFH uptake was not obvious (Figure 3A). Therefore, replacing cholesterol with RG3 could effectively reduce foreign body phagocytosis by RAW264.7 cells. It is evident that the hydrophilic surface hydration layer on RG3 may affect the biological activity of the nanoprobes (Dadashzadeh et al., 2010; Hadjidemetriou et al., 2015). As an amphiphilic structural component of the membrane, RG3 reduces the effects of the circulating protein corona (Xia et al., 2023), which is highly susceptible to formation on a variety of cationic nanosurfaces. Although numerous studies have been devoted to modifying these surfaces with targeting ligands, such as RGD peptides, to enhance drug uptake in the target region (Katsamakas et al., 2017), the targeting ligand may be masked by the protein corona. Therefore, we designed our nanoprobe to have a negatively charged surface to reduce the nonspecific adsorption of circulating proteins, thereby avoiding clearance by the reticuloendothelial system and achieving long circulation times and high target aggregation in synergy with the stealth polyethylene glycol nanocarrier.

Further observations revealed that both HMME-CHOL@PFH and HMME-RG3@PFH could be taken up by KFs, but the intracellular fluorescence (representing binding) in the HMME-RG3@PFH group was significantly greater than that of the HMME-CHOL@PFH group (Figure 3B). These data indicated that RG3 could better target and recognize KFs which was mainly related to the GLUT1-mediated endocytosis pathway in addition to passive targeting by the EPR effect (Hong et al., 2019). RG3 on the surface of the lipid nanomembrane binds well to GLUT-1 overexpressed in KFs via its hydrophilic glucose side chains to enhance targeted drug uptake. Moreover, this transporter protein was found to be upregulated in numerous cancers (Zhu et al., 2021). Therefore, the specific binding of nanoprobes to transporter proteins enhances target-area aggregation. KFs exhibited time-dependent uptake of the DiD-labeled HMME-RG3@PFH nanohybrid, with nonsignificant intracellular red fluorescence after 1 h of incubation and an increase in intracellular fluorescence at 2 h, and a large amount of uptake at 4 h (Figure 3C). In contrast, no significant uptake of HMME-RG3@PFH was observed in HUVECs (Figure 3D). Quantitative analysis by flow cytometry indicated that after 4 h of incubation, more than 95.08% of the nanomaterial was taken up by KFs (Figure 3E), which was significantly greater than the uptake of HMME-RG3@PFH by HUVECs under the same conditions (9.3%, Figure 3F). This was attributed to the overexpression of GLUT-1 on the surface of the KF cell membrane. In some hypoxic environments, KFs overexpress relevant glucose transporters to meet the demands of high cellular metabolism (Wang et al., 2021; Temre et al., 2022), while glucose protein transporters can affect cellular metabolism through the glycolytic pathway (Vinaik et al., 2020).

### 3.4 SDT effect

**FIGURE 4:**
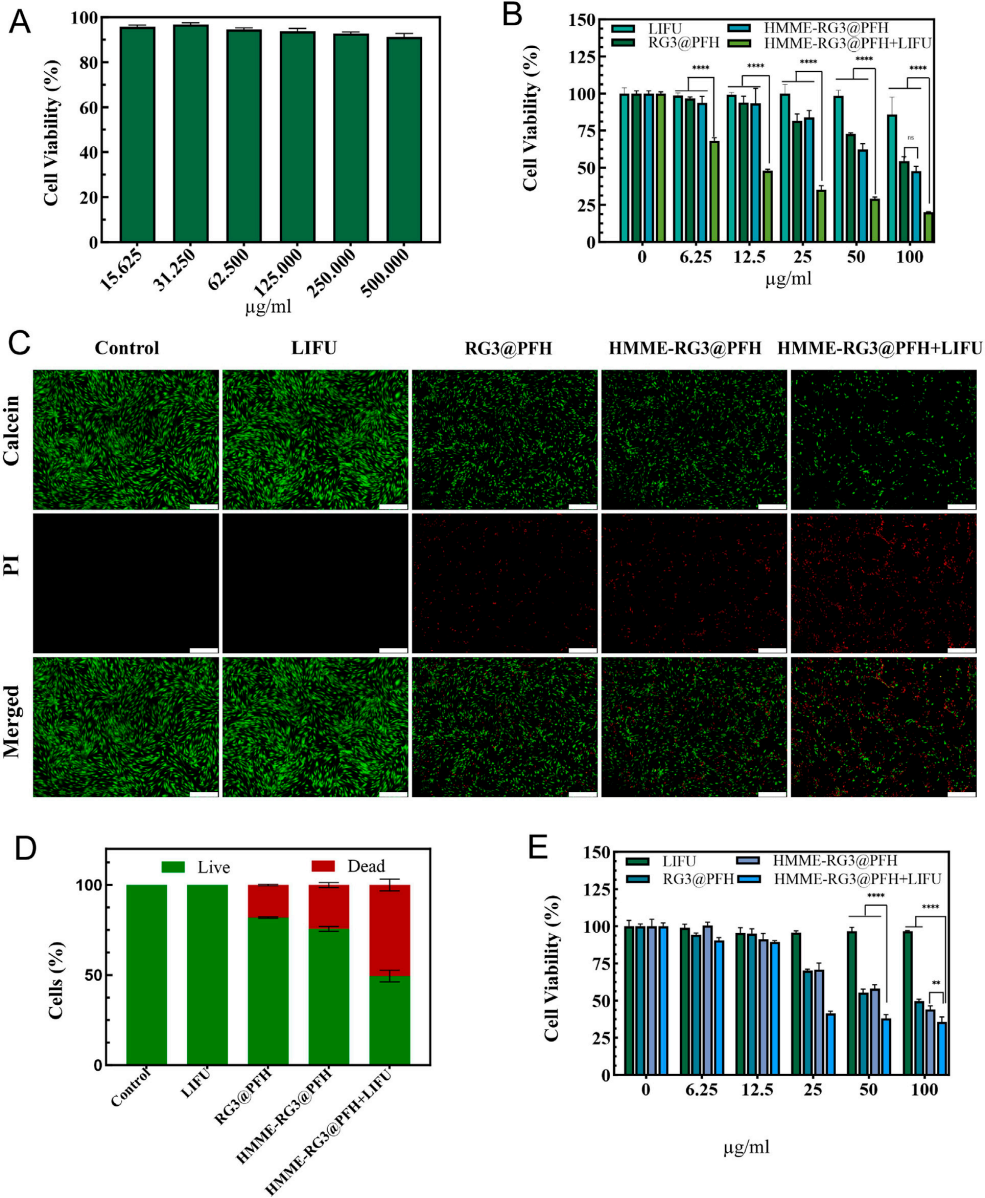
SDT efficacy of HMME-RG3@PFH. **(A)** Activity of KFs treated with different concentrations of blank nanocomposites. **(B)** Survival of KFs. **(C)** Calcein-AM/PI staining images of KFs under different experimental conditions. Live cells are shown in green and dead cells are shown in red; scale bar: 500 μm. **(D)** Calcein-AM/PI fluorescence staining of KFs. **(E)** Survival of HUVECs. (n = 3, ****p < 0.0001, **p < 0.01).

To investigate the therapeutic effect of HMME-RG3@PFH combined with LIFU on KFs, the effect of blank lipid nanoparticles on the proliferation of KFs was first detected, and cell viability reached 90% at a concentration of 500 μg/mL (Figure 4A). Afterward, the effects of different experimental conditions on KFs were explored, and the KFs were randomly divided into the LIFU, RG3@PFH, HMME-RG3@PFH, and HMME-RG3@PFH + LIFU groups. A dose-dependent inhibitory effect on KFs was observed with each nanomaterial, which was related to the encapsulated RG3 component. By regulating the TGF-β/Smads and ERK pathways, RG3 inhibited fibroblast proliferation (Tang M. et al., 2018). There was no significant difference in cell proliferation between the RG3@PFH group and the HMME-RG3@PFH group, indicating that HMME alone was not sufficient to affect fibroblast proliferation. Inhibition in these groups remained less than 50% even when the concentration of the materials reached 100 μg/mL. However, HMME-RG3@PFH in combination with LIFU demonstrated a strong inhibitory effect, as KF survival was only 35% at 25 μg/mL (Figure 4B). Calcein-Am/PI staining clearly showed that the signal intensity of the group treated with LIFU alone did not differ from that of the control group, and no red fluorescence was observed. Little red fluorescence was observed in the two drug-loaded nanoparticle groups, HMME-RG3@PFH and RG3@PFH, indicating that RG3 could regulate KFs to a certain extent, but the signal intensity of HMME-RG3@PFH was significantly enhanced when it was applied in combination with LIFU irradiation (Figures 4C, D). HMME-RG3@PFH gradually expanded under LIFU irradiation, and after reaching the acoustic pressure threshold, the targeted destruction of the microbubbles exposed the HMME and more hydrophobic RG3 fragments. Then, HMME produced an intracellular “blast effect” for SDT, which led to a significant decrease in the activity of KFs; on the other hand, RG3 stabilized the KFs after SDT, ultimately achieving synergistic sensitization under LIFU and effectively reducing fibroblast density.

A complex regulatory mechanism exists between fibroblasts and vascular endothelial cells, as the excess of newly generated immature blood vessels provide nutritional support for fibroblasts (Mogili et al., 2012)while the activated fibroblasts produce VEGF to promote the abnormal proliferation of endothelial cells (Nagy et al., 2008). Moreover, the excessive proliferation and contraction accelerate vascular occlusion and reduce tissue vascular patency. Therefore, remodeling the peripheral vasculature to ameliorate this excessively aggregated state is particularly necessary. HUVECs, which aim to remodel the cellular microenvironment by inhibiting the microvasculature, were inhibited more weakly than were KFs at a HMME-RG3@PFH concentration of 12.5 μg/mL, which was attributed to the efficient uptake of these nanoparticles by KFs. Increasing the concentration to 25 μg/mL significantly enhanced the effects of both RG3@PFH and HMME-RG3@PFH in HUVECs (Figure 4E). RG3 downregulated angiogenic gene expression, which in turn blocked angiogenesis (Tang YC. et al., 2018), and HMME-RG3@PFH combined with LIFU inhibited HUVECs more strongly. After being combined with LIFU-induced microbubble rupture, the vascular structural gaps widened, affecting the cytoskeleton and cellular membrane permeability. This further led to apoptosis and necrosis, thus reducing the aberrations in the microvessels in the target area.

### 3.5 Cell migration and invasion

**FIGURE 5:**
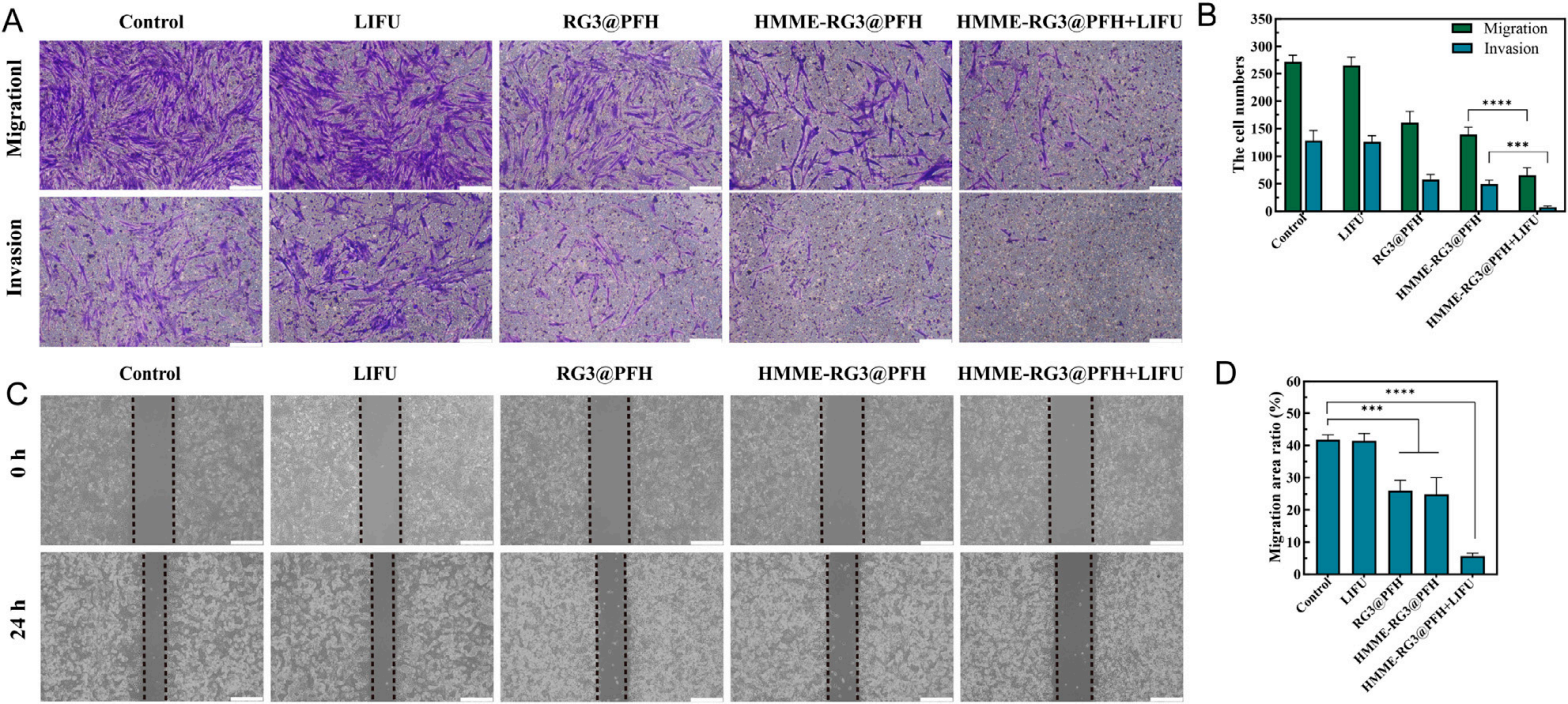
Cell migration and invasion analyses. **(A)** Number of KFs in the lower chamber of the transwell; Scale bar: 200 μm. **(B)** Quantitative analysis of KF migration and invasion. **(C)** Comparison of the migration of scratched HUVECs at 0 h and 24 h; Scale bar: 200 μm. **(D)** Comparison of HUVEC migration rates. (****p < 0.0001, ***p < 0.001).

Activated fibroblasts are very aggressive, and they promote tumor growth while impeding drug delivery by secreting protumor cytokines and increasing mesenchymal pressure (Guo et al., 2020). Therefore, it is particularly important to regulate cellular activity to promote drug penetration. To further evaluate the effect of the drug-loaded nanoparticles on cell activity, KF migration was detected via Transwell assays, and no cell inhibition was detected by LIFU irradiation alone. Compared with the control group, the drug-loaded group inhibited the migration of KFs to a certain extent (p < 0.05), but no significant difference was detected between the RG3@PFH group and the HMME-RG3@PFH groups. Moreover, the HMME-RG3@PFH group demonstrated strong KF inhibition when used in combination with LIFU, which ultimately led to a significant reduction in the number of cells that migrated to the bottom chamber (Figures 5A, B). These results indicate that the nanoprobe HMME-RG3@PFH is a promising therapeutic agent, and the regulation of fibroblasts by HMME via SDT combined with RG3 resulted in a significant decrease in their activity, which also highlights the effectiveness of ADV-mediated targeted therapy. Such activated fibroblast-based therapy, which involves remodeling of the cellular “soil environment” to restore fibrotic matrix homeostasis through cellular phenotype reversal, can fully enhance the efficacy of drug therapy (Han et al., 2018).

Moreover, the microvascular effects of HMME-RG3@PFH were visually evaluated had this nanomaterial had a certain effect on HUVEC migration, which showed a 24 h migration rate of only 6% under the combined effects of LIFU (Figures 5C, D). This was significantly lower than those in the control and LIFU alone groups, which was attributed to the angiogenesis-disrupting effect of RG3 (Tang M. et al., 2018). The cytostatic effect was further amplified by LIFU irradiation, demonstrating that HMME-RG3@PFH has a synergistic potential under LIFU irradiation, that the nutrient supply to KFs can be blocked by inhibiting angiogenesis, and that some of the methods of embolizing the tumor vasculature via initialization of the coagulation synergistic amplification are also essential for destroying vascular endothelial cells to achieve therapeutic goals (Xie et al., 2022; Ho and Yeh, 2017). Additionally, the ability of ADV to induce vascular rupture and enhance drug penetration in solid tumors has been demonstrated (Ho et al., 2016; Zhu et al., 2018).

### 3.6 Intracellular ROS

**FIGURE 6:**
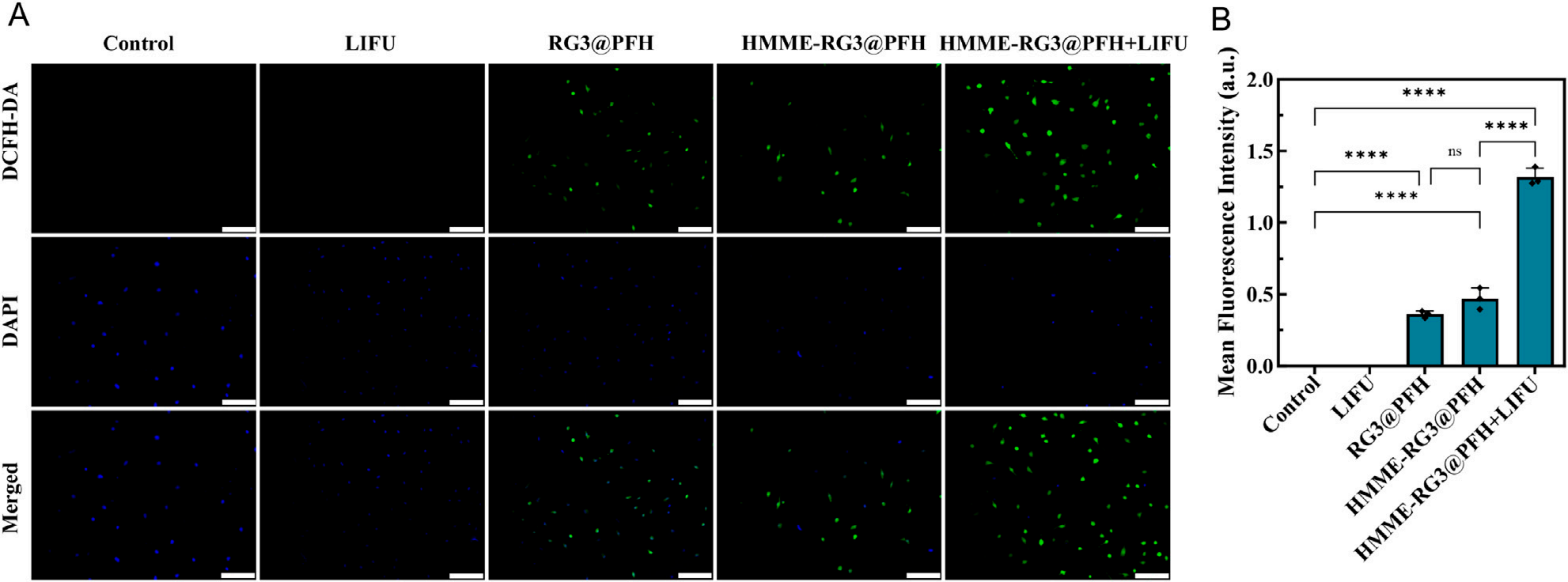
ROS enhancing effect of HMME-RG3@PFH. **(A)** Image of the fluorescence intensity of ROS in KFs after treatment; scale bar: 200 µm. **(B)** Analysis of ROS fluorescence intensity in KFs.

Mitochondria are the main targets of cellular oxidative stress, and cytotoxic ROS can lead to organelle dysfunction and induce programmed cell death. Notably, compared with those in the control, LIFU, RG3@PFH, and HMME-RG3@PFH groups, significant green fluorescence from ROS was detected in the HMME-RG3@PFH + LIFU group (Figures 6A, B), while no fluorescence signal was detected in the cells treated with LIFU alone, suggesting that LIFU irradiation alone does not produce ROS. Moreover, a little green fluorescence was detected in the KFs after treatment with HMME-RG3@PFH and RG3@PFH and there was no significant difference between the groups, suggesting that modified RG3 can regulate intracellular ROS to exert an inhibitory effect (Hwang et al., 2022). Moreover, HMME accumulation alone does not affect intracellular ROS levels, as the green fluorescence intensity of ROS can further increase only under LIFU irradiation due to the powerful acoustic catalytic effect of HMME under LIFU irradiation (Xi et al., 2022), which ultimately increased the ROS production rate by nearly threefold. An imbalance in the intracellular redox system due to SDT can exacerbate intracellular toxicity, and radiotherapy induces ROS production, leading to apoptosis (Brenneisen and Reichert, 2018). However, it is difficult to achieve the expected efficacy within some solid tumors due to the antioxidant system in the tumor cells and the immune tolerance mediated by fibroblasts. Thus, increasing SDT sensitivity in the target area by interfering with fibroblasts enhances the efficacy of cellular combination therapy.

### 3.7 Hemolysis test

**FIGURE 7:**
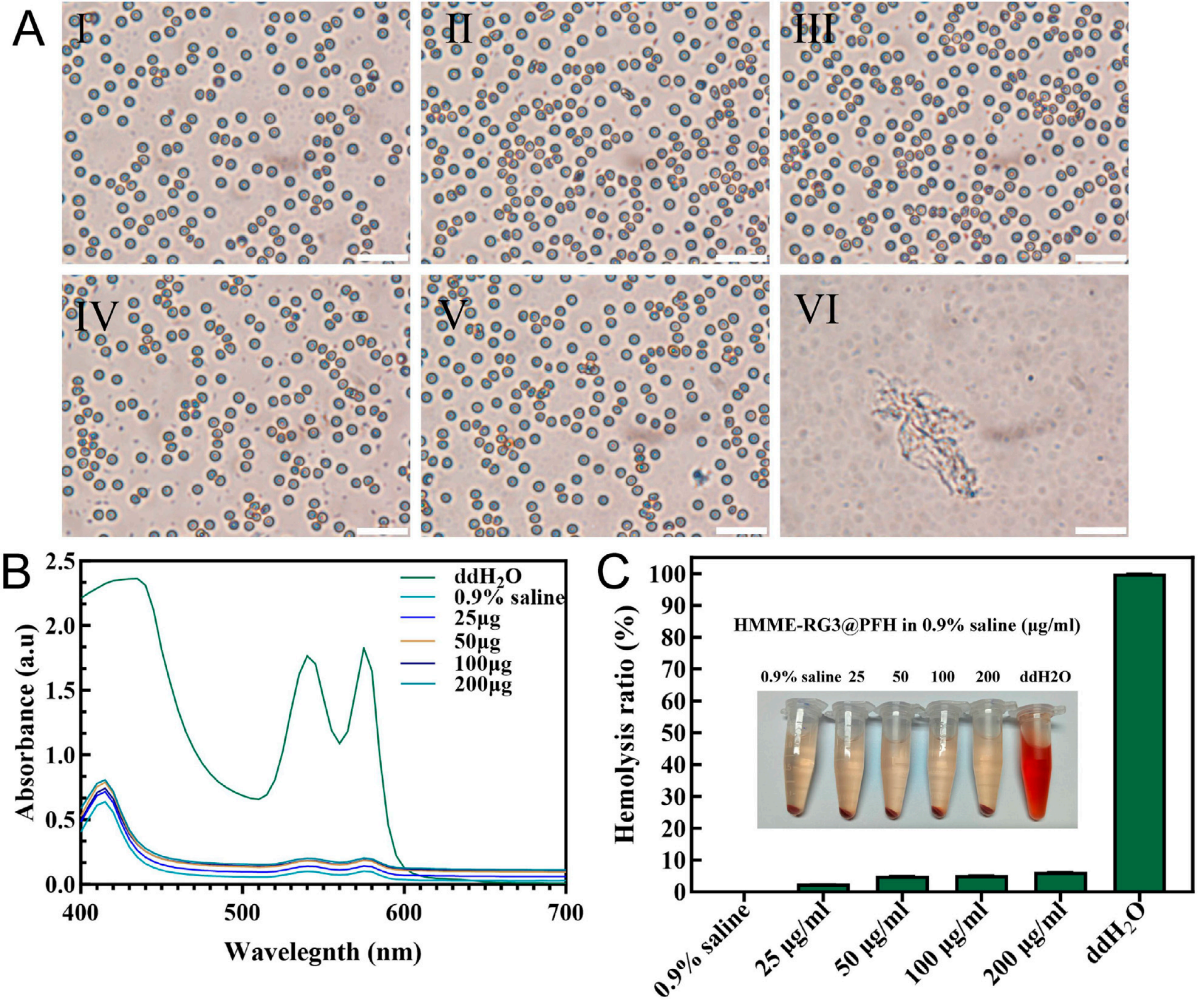
Analysis of blood cell hemolysis after coincubation with HMME-RG3@PFH. **(A)** Microscopic morphology of erythrocytes after 4 h of coincubation (I: 0.9% saline; II-V: nanomaterial solutions at concentrations of 25 μg/mL, 50 μg/mL, 100 μg/mL, and 200 μg/mL, respectively; and VI: ddH_2_O; scale bar: 50 µm). **(B)** UV absorption curves of hemoglobin from each group. **(C)** Hemolysis rates and visual observations from the different experimental groups.

The biosafety of HMME-RG3@PFH was evaluated using a hemolysis assay. Under the microscope, the erythrocytes in the ddH_2_O group had aggregated into clusters after cleavage, while no obvious hemolysis was detected in the HMME-RG3@PFH group, and the erythrocytes in solutions of nanomaterials at various concentrations maintained their biconcave discoid shape and homogeneous distribution (Figure 7A). The ddH_2_O group had the strongest UV absorption value after coincubation with red blood cells (RBCs) at 540 nm, while the absorption values in all of the other groups were lower (Figure 7B). Moreover, no obvious hemolysis was observed even at 200 μg/mL HMME-RG3@PFH, as the solution was a light orange color, and the corresponding hemolysis rate was only 6% (Figure 7C). These data show that the lipid nanoprobe HMME-RG3@PFH possessed good biosafety. The experimental nanocarrier adopts a core-shell structure with a phospholipid core, which is suitable for loading amphiphilic drug molecules and broadens its prospects of clinical application. Moreover, HMME-RG3@PFH has a membrane structure similar to that of the cells of living organisms, which gives it excellent biocompatibility. Thus, lipid envelopes have been chosen as the delivery medium for emerging mRNA therapeutics (Qiu et al., 2021; Verbeke et al., 2022; Wang et al., 2023), which effectively reduces drug degradation during transportation and improves the cellular uptake of the vaccine mRNA, showing that lipid materials have great potential for future clinical applications.

### 3.8 *In vivo* inhibition

#### 3.8.1 Visible assessment

**FIGURE 8:**
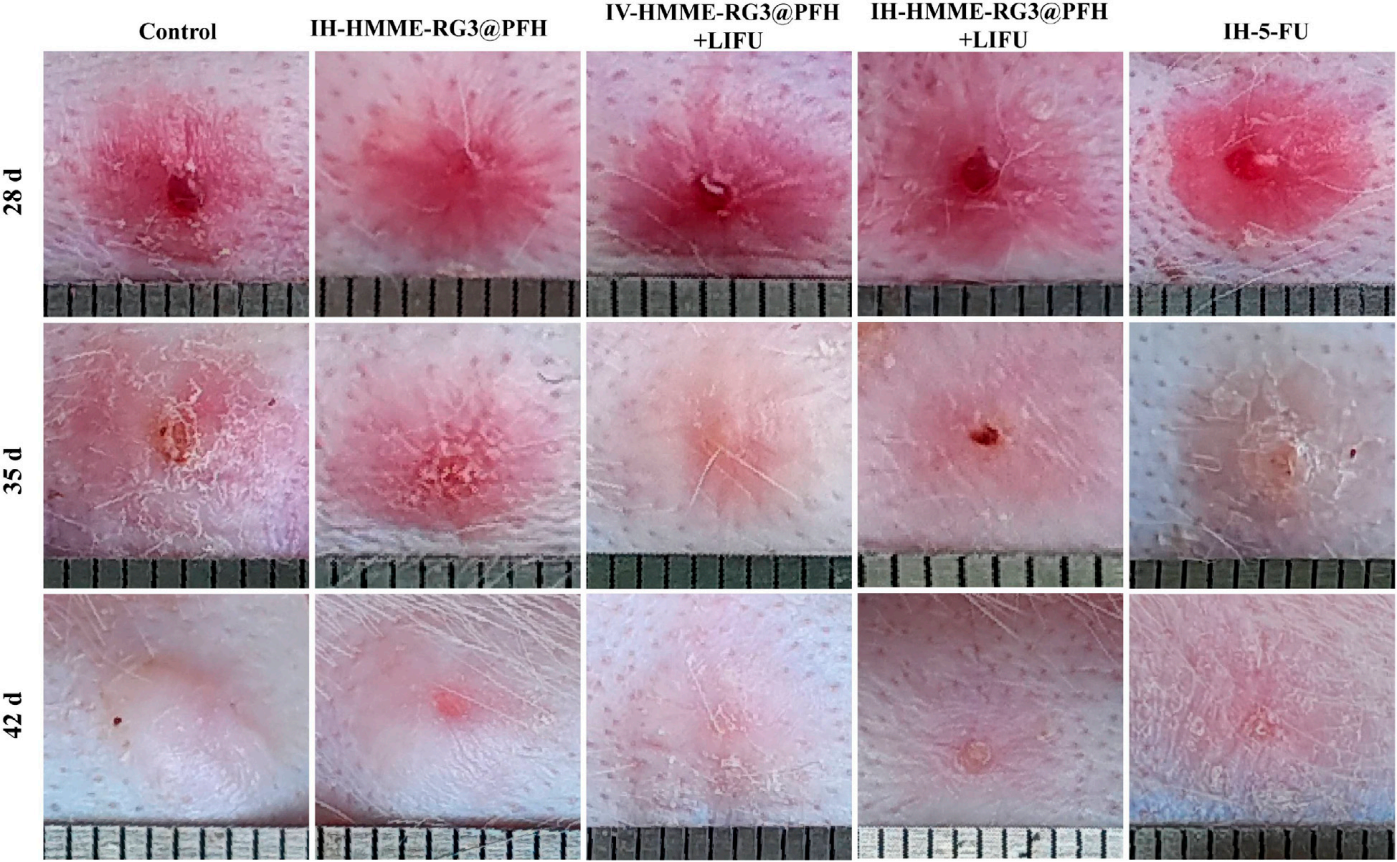
Tissue proliferation observed at 28, 35 and 42 days after surgery. The IH-HMME-RG3@PFH, IH-HMME-RG3@PFH + LIFU, IV-HMME-RG3@PFH + LIFU and IH-5-FU groups received local injection of HMME-RG3@PFH, local injection of HMME-RG3@PFH + LIFU, intravenous injection of HMME-RG3@PFH + LIFU and local injection of 5-FU, respectively.

Next, we established a pathological rabbit ear scar model to observe and compare fibroblasts under different treatments and evaluate fibroblast inhibition by the nanoprobe HMME-RG3@PFH in combination with LIFU. First, we visually evaluated the effect of the drug-carrying nanoprobe on the fibroblasts in the scars by the naked eye. On the 28th day after the operation, all the wounds were completely epithelialized, the skin surfaces were red, and stiff and raised scar tissue had developed (Figure 8). Afterward, the rabbits were randomly divided into the following groups and treated accordingly: control, IH-HMME-RG3@PFH, IH-HMME-RG3@PFH + LIFU, IV-HMME-RG3@PFH + LIFU, and IH-5-FU. Thirty-five days after surgery, local reddening of the tissue was observed in each group, which was related to the reactive wound stimulation caused by local injection. The scarring in the IH-HMME-RG3@PFH + LIFU treatment group was largely improved with results similar to those in the 5-FU group, and both of these groups exhibited more superficial scarring than the control group. Notably, tissue scarring in the IV-HMME-RG3@PFH + LIFU group did not change as much as that in the IH-HMME-RG3@PFH + LIFU group, but the IV-HMME-RG3@PFH + LIFU group had a smaller wound reaction and smoother local tissues. Moreover, the therapeutic effect in the IH-HMME-RG3@PFH group was less pronounced, and localized tissue elevation remained, while there was marked elevation in the control group. These results demonstrated that intravenously injected nanoparticles more uniformly targeted and aggregated locally in the lesion, producing the satisfactory result of smoother scar. But, IV-HMME-RG3@PFH + LIFU treatment was not as effective as IH-HMME-RG3@PFH + LIFU due to insufficient nanoparticle concentration in the target area of long-circulation action. Therefore, the next step of the study could be further optimized in terms of increasing both nanoparticle loading and the duration of circulation to reduce damage.

At 42 days after surgery, the skins of all the rabbits in the experimental groups showed some degree of whitening. Moreover, the surface morphology in the IH-HMME-RG3@PFH + LIFU group was significantly improved and similar to that in the IH-5-FU group, which exhibited a flatness consistent with that of the surrounding normal tissue. In contrast, the tissue height of the IV-HMME-RG3@PFH + LIFU group had decreased, but there was still slight elevation. In the control and IH-HMME-RG3@PFH groups, a whiter tissue color was observed, which was accompanied by elevation and local tissue shrinkage, especially after re-epithelialization, and the texture of these tissues was significantly harder. Taken together, the above observations indicated that under LIFU irradiation, the nanoprobe HMME-RG3@PFH targeted drug release, which effectively inhibited the abnormal proliferation of fibroblasts through the fixed point bursting mechanism, and that the synergistic treatment modality involving local injection of HMME-RG3@PFH and LIFU irradiation significantly enhanced fibroblast inhibition compared with local injection of HMME-RG3@PFH alone. This advantage was mainly attributed to the inhibitory effect of HMME under acoustic activation, and when combined with the regulation by RG3 *in vivo*, the cytostatic effect of HMME-RG3@PFH was further enhanced.

#### 3.8.2 Changes in histologic characteristics

**FIGURE 9:**
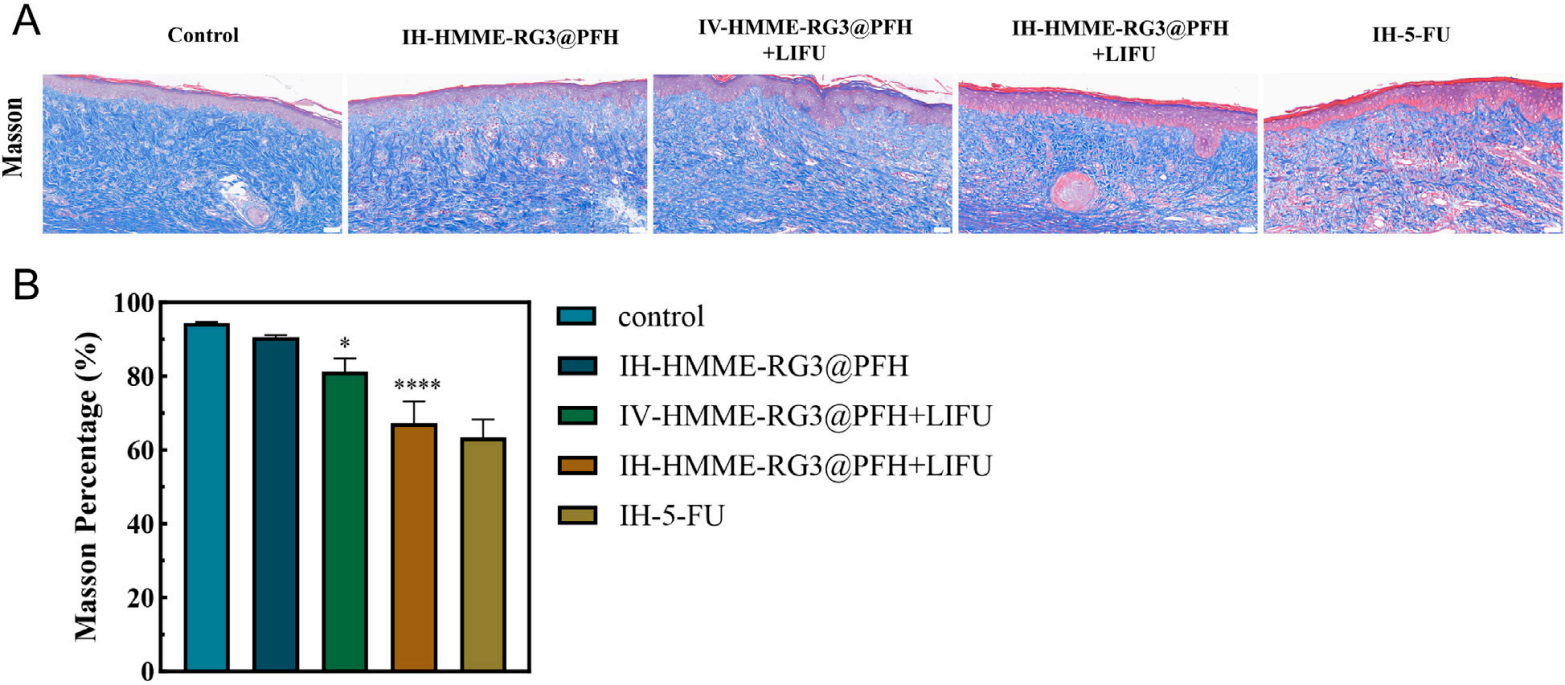
**(A)** Masson staining images of scar tissue sections taken 49 days after surgery; scale bar: 50 µm. **(B)** Analysis of masson staining in scar tissue.

Trauma histology is important for analyzing the inhibition of activated fibroblasts. ECM collagen deposition is the main pathological manifestation of abnormal fibroblast activation; therefore, Masson staining was used to observe the microstructural features of the tissue. Masson staining revealed that at 49 days after surgery, IH-HMME-RG3@PFH in combination with LIFU significantly improved collagen fiber deposition and the collagen was more loosely arranged, showing a therapeutic effect similar to that of 5-FU injection, while dense and disorganized collagen fibers were observed in the injured tissues of the control group (Figure 9). Moreover, we found that IV-HMME-RG3@PFH + LIFU treatment was not as effective as IH-HMME-RG3@PFH + LIFU treatment after analysis of the complex *in vivo* microenvironment. The reason for this may be due to the relative decrease in uptake due to metabolic losses via the circulation, whereas maximum uptake can be achieved by direct, local intratumoral injection; nonetheless, some large or deep tissue-activated fibroblasts cannot be treated by local drug administration. Notably, after combination with LIFU irradiation, the HMME-RG3@PFH nanoprobe demonstrated significant therapeutic effects through acoustic power synergistic amplification, showing excellent inhibition of abnormally proliferating fibroblasts, which in turn improved the relevant pathological state. These results strongly support the application of the HMME-RG3@PFH nanoparticles in the treatment of relevant diseases.

## 4 Conclusion

In this work, a novel targeted lipid nanoprobe, HMME-RG3@PFH, with an acoustic kinetic therapy mechanism, was successfully prepared. The physicochemical properties of the nanoprobe were as expected, indicating its good biosafety.

HMME-RG3@PFH effectively targets MFs and has a superior immune escape ability from macrophages, resulting in a long circulation time.

HMME-RG3@PFH demonstrates excellent dual-modal imaging capabilities for the early diagnosis of MF-related diseases and can be further used for visualizing therapeutic treatments and precise drug release.

Relevant *in vivo* and *in vitro* experiments confirmed that HMME-RG3@PFH has a cascading regulatory effect on MFs, which provides a new pathway for the treatment of MF-related diseases.

## Data Availability

The raw data supporting the conclusions of this article will be made available by the authors, without undue reservation.
